# Recent trends in movement ecology of animals and human mobility

**DOI:** 10.1186/s40462-022-00322-9

**Published:** 2022-05-25

**Authors:** Rocío Joo, Simona Picardi, Matthew E. Boone, Thomas A. Clay, Samantha C. Patrick, Vilma S. Romero-Romero, Mathieu Basille

**Affiliations:** 1 Department of Wildlife Ecology and Conservation, Fort Lauderdale Research and Education Center, University of Florida, Fort Lauderdale, FL USA; 2 Global Fishing Watch, Washington DC, USA; 3 Jack H. Berryman Institute and Department of Wildland Resources, S.J. & Jessie E. Quinney College of Natural Resources, Utah State University, Logan, UT USA; 4 School of Environmental Sciences, University of Liverpool, Liverpool, UK; 5 Institute of Marine Sciences, University of California Santa Cruz, Santa Cruz, CA USA; 6 Systems Engineering, Faculty of Engineering and Architecture, University of Lima, Lima, Peru

**Keywords:** Biologging, Movement ecology framework, Tracking technology, Text mining, Interdisciplinarity

## Abstract

Movement is fundamental to life, shaping population dynamics, biodiversity patterns, and ecosystem structure. In 2008, the movement ecology framework (MEF Nathan et al. in PNAS 105(49):19052–19059, 2008) introduced an integrative theory of organismal movement—linking internal state, motion capacity, and navigation capacity to external factors—which has been recognized as a milestone in the field. Since then, the study of movement experienced a technological boom, which provided massive quantities of tracking data of both animal and human movement globally and at ever finer spatio-temporal resolutions. In this work, we provide a quantitative assessment of the state of research within the MEF, focusing on animal movement, including humans and invertebrates, and excluding movement of plants and microorganisms. Using a text mining approach, we digitally scanned the contents of $>8000$ papers from 2009 to 2018 available online, identified tools and methods used, and assessed linkages between all components of the MEF. Over the past decade, the publication rate has increased considerably, along with major technological changes, such as an increased use of GPS devices and accelerometers and a majority of studies now using the R software environment for statistical computing. However, animal movement research still largely focuses on the effect of environmental factors on movement, with motion and navigation continuing to receive little attention. A search of topics based on words featured in abstracts revealed a clustering of papers among marine and terrestrial realms, as well as applications and methods across taxa. We discuss the potential for technological and methodological advances in the field to lead to more integrated and interdisciplinary research and an increased exploration of key movement processes such as navigation, as well as the evolutionary, physiological, and life-history consequences of movement.

## Main text

**Fig. 1:**
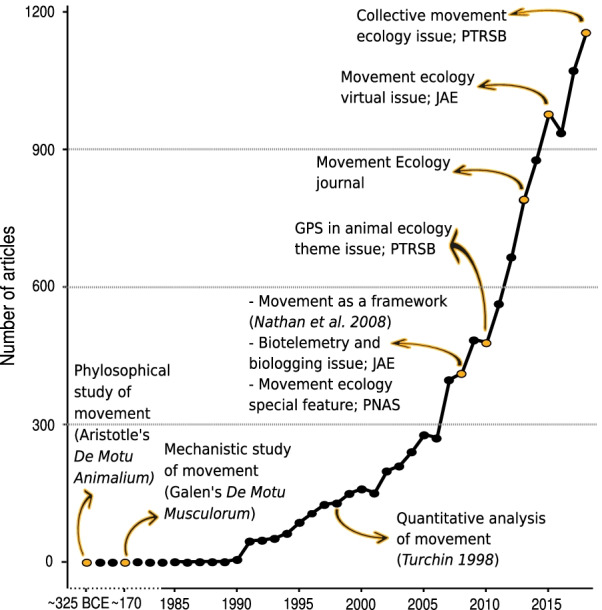
Number of articles published each year until 2018 in movement ecology of animals and human mobility as identified by our algorithm, along with a timeline of key movement papers and milestones in the field. PNAS: Proceedings of the National Academy of Sciences; JAE: Journal of Animal Ecology; PTRSB: Philosophical Transactions of the Royal Society B. See alt-text in the Alt-text section of the manuscript

Movement, defined as a change in position of an individual in time, has been studied at least since classical antiquity, both from conceptual (Aristotle’s *De motu animalium* 384-322 BC) and mechanistic (Galen’s *De motu musculorum* 129-210 AD) perspectives (Fig. 1). Since then, many conceptual and technological innovations have contributed to shaping the field that today we call movement ecology [1, 2]. Conceptually, movement began to draw attention in modern ecology because of its implications for reproduction, gene flow, and metapopulation dynamics; as such, studies on animal and plant dispersal constituted a large portion of the ecological literature on movement until the early 2000s [3–5]. Migration also historically constituted an important focus—with notable theoretical advances stemming from research on insects [6, 7]. The study of wildlife migration in the field was revolutionized by the invention of bird banding, famously used by John James Audubon in the 1800s but appearing in history records as far back as the 1600s [8]. A few centuries later, the first radio-telemetry devices were deployed in the 1960s on wild porcupines (*Erethizon dorsatum*) by Marshall et al. [9], soon followed by studies on grizzly bears (*Ursus arctos horribilis*) by the Craighead brothers [10, 11] and on various mesomammals by Cochran and Lord Jr [12]. Approximately at the same time, Kooyman [13] pioneered the use of animal-borne sensors in marine mammals. Bird banding and telemetry opened the way for scientists to measure wildlife movement from a Lagrangian perspective—i.e. tracking movements of a single individual through time, as defined by Turchin [14], who borrowed terminology from fluid dynamics to describe organismal movement in his foundational book.

In 2008, Nathan et al. [1] proposed the movement ecology framework (MEF) to unify movement research. The MEF intended to develop ‘an integrative theory of organism movement for better understanding the causes, mechanisms, patterns, and consequences of all movement phenomena’ [15]. To this aim, the MEF focused on the links between four components: internal state (why move?), navigation capacity (where to move?), motion capacity (how to move?), and external factors (the set of biotic and abiotic environmental factors that affect movement). While several studies already existed that addressed the interplay between internal state and external factors in determining the emergence of movement [16], the goal of the MEF was to formalize links between factors affecting movement and favor integration in the years to come. Technology allowing us to track individuals for long periods of time and at fine scales, as well as methodologies to infer behaviors from movement patterns and link them to motion and navigation capacities, internal characteristics, and external factors, were listed as main requirements and challenges to quantify the movement of individuals within the new integrative framework [1].

Technological advancements have since powered an exponential expansion of the field of movement ecology. Cagnacci et al. [17] defined the development of GPS-tracking technology as ‘a perfect storm of opportunities’ for the study of animal movement. Loggers have become smaller, cheaper, and more reliable, allowing for more animals to be tagged, for data to be collected at ever finer spatio-temporal resolutions [18], while uncovering previously unknown and unattainable behaviors in wildlife [2]. Wilmers et al. [19] coined the term ‘golden era of biologging’ to describe this recent period, as the widespread diffusion of a variety of animal-borne sensors (including but not limited to GPS devices, accelerometers, magnetometers, cameras, etc.) continues to open new and exciting possibilities for the study of wildlife movement.

Modern movement literature places itself at the interface of several research fields, including physics [20], physiology [21], data science [22], and ecology [23]. The development and widespread use of tracking devices is simultaneously propelling human mobility science [24], a discipline that has borrowed several concepts and approaches from animal studies, due to the latter’s longer history investigating movement from telemetry data [25]. Data on human mobility is often quantified and analyzed using collective or Eulerian approaches [26, 27], which in turn could be beneficially incorporated into animal movement ecology studies. Indeed, initiatives for reciprocal integration of both animal movement and human mobility have already started [26, 27].

In recent years, the technological and analytical advances for animal and human tracking triggered the emergence of a series of reviews related to sensors, software, and statistical and mathematical tools to study different aspects of movement ecology [2, 25, 27–29]. We deemed it timely to complement these reviews with a quantitative assessment of movement research in animals—including humans—in 2009–2018, a full decade after the publication of the MEF. While some human mobility studies have proposed perspectives and frameworks alternative to the MEF [30, 31], the MEF is compatible with other frameworks [27] and we chose to focus on it for understanding movement. Using a text mining approach, we were able to collect data from a large number of peer-reviewed publications in animal movement; thus including humans and invertebrates, and excluding movement of plants and microorganisms. We investigated general trends in the topics studied, the use of tracking devices, software, statistical methods, the studied taxonomic groups, and the components of the MEF. In light of our findings, we provide recommendations for future directions to ensure that technological advancements improve our understanding of movement.

## Materials and methods

### Data collection

For the purpose of this review, we searched for scientific peer-reviewed papers in English that studied the voluntary movement of animals or humans. In order to highlight relevant trends in the literature, we extracted a large sample of papers studying animal and human movement from the full scientific literature. Very few papers explicitly mentioned ‘movement ecology’ in their abstracts, titles and keywords, so we could not simply use ‘movement ecology’ as a search phrase. Instead, we designed an iterative extraction algorithm that ensured we would obtain (1) a high proportion of sampled papers studying animal or human movement and (2) a sample large enough to explore trends in many dimensions. In terms of model performance, the first criterion was akin to achieving high precision, i.e. the proportion of sampled papers (predicted to be of animal or human movement) that actually correspond to animal or human movement, for which we fixed a minimum threshold of 0.8. The second criterion was fulfilled with a sample in the order of thousands.

**Fig. 2:**
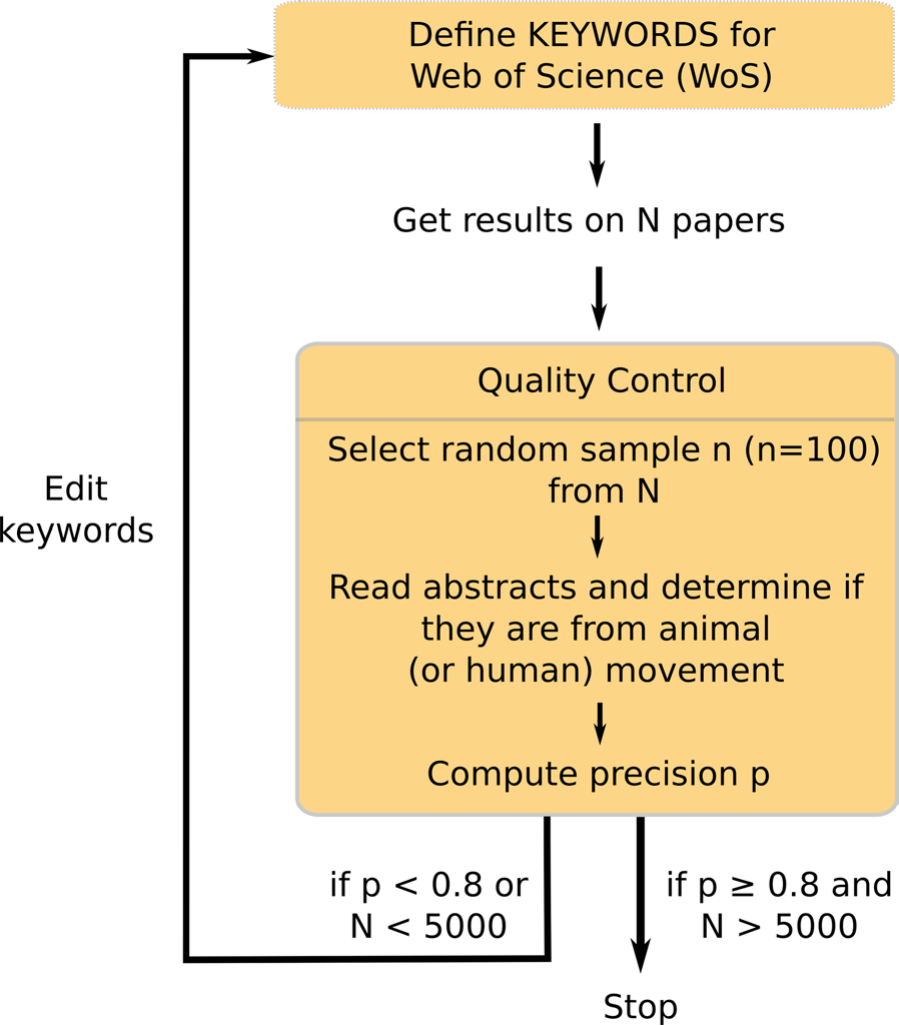
Graphical representation of the algorithm to identify animal movement papers (including human mobility) described in the Data collection section. See alt-text in the Alt-text section of the manuscript

For practical reasons as well as the sheer size of their database, the Web of Science (WoS) was the search engine used. The iterative algorithm worked as follows (Fig. 2):

1. Define (or refine) groups of keywords;
2. Use keywords on WoS and extract the results (*N* papers);
3. Perform quality control:
  - Select a random sample (*n* = 100) from *N*;
  - Read abstracts and determine if they fall within our definition of “animal and/or human movement”;
  - Compute precision *p*, the proportion of true abstracts within the scope;
  - Identify which words triggered false positives;
4. Repeat all the previous steps until $p>0.8$. *p* may come at the expense of N, so we also required that *N* would be in the order of thousands (we set a cut-off of at least 5000 papers).

After several rounds of testing, we came up with the following four groups of words:

- *Group 1: Behavior* behavio
- *Group 2: Biologging* animal-borne, accelerom, argos, biologg, bio-logg, geolocat, geo-locat, gls, gps, radar, reorient, sonar, telemetry, vhf, vms
- *Group 3: Individuals* animal, fishermen, human, individual, people, person, player, wildlife
- *Group 4: Movement* kinematics, motion, movement, moving, spatio-temporal, spatiotemporal

Abstracts were selected if they included words from at least 3 of the 4 groups above. For abstracts that included words from groups 1, 3, and 4, we further identified a set of keywords to discriminate papers outside of our scope (i.e., papers that did not focus on the voluntary movement of animals or humans). As such, unless they had words from group 2, we excluded papers with the following words in the abstract:

- *Group 5: Excluded words* atom, cell, cortex, cortic, counsel, cognit, DNA, enzyme, eye, insulin, lymph, market, molecule, neurons, neurotransmi, particle, patient prosthese, spine, questionnaire, sedentary, strain, tectonic

The search was run on WoS in November 2019 over 273 journals (available in the Additional file 1 at https://rociojoo.github.io/mov-eco-review/posts/html/Journal_table_3col.html). The selection of journals was made in parallel with keyword tuning, i.e., while executing the search algorithm, we looked at the list of journals where the papers were published, and those with an irrelevant scope (e.g., fashion) or that were not peer-reviewed were filtered out.

Precision of the final algorithm was equal to 0.90; i.e. 90 out of 100 papers detected by the algorithm were correctly identified as animal or human movement papers based on manual verification. This high precision indicates that there is a high certainty that those retained are in fact movement papers. Additionally, we wanted to obtain a recall or sensitivity rate to quantify, from all movement papers in the literature, how many we had in our search results. As it is impossible to obtain the complete list of animal and human movement papers in the literature, we instead looked at the list of papers published by the journal Movement Ecology. We found that $69\%$ were in our list. Considering that we excluded research on plant movement ecology from our search, this percentage seems reasonably high.

### Data processing

The results from WoS included fields such as title, abstract, keyword, authors, and others. We used the refsplitr package [32] in the R software [33] to read the files downloaded from WoS and compile them into one data sheet.

Depending on the scope of the paper, it is possible that the tracking devices, software, or statistical methods used are not mentioned in the title, abstract, or keywords of the paper, but only in the material and methods (M&M) section (Table 1). To obtain the M&M sections, we downloaded all manuscripts using the fulltext R package [34], and the API keys from Elsevier, Springer, Scopus, Wiley, BMC, and PLOS One. Then, we created codes to extract M&Ms depending on whether the files were in xml or pdf format. More detailed descriptions of the procedures used to extract the M&Ms sections can be found in Additional file 1: Sect. 2.3 at https://rociojoo.github.io/mov-eco-review/data-collection-and-processing.html#extracting-the-material-and-methods-mm-sections, with links to the code files therein.

**Table 1:** Sections of articles in movement ecology of animals and human mobility used for each dimension analyzed in the current study

Dimension	Title	Keywords	Abstract	M&M
Taxonomy	X	X	X	
Devices	X	X	X	X
Software	X	X	X	X
Methods	X	X	X	X
Framework	X	X	X	
Topics			X	

### Dictionary approach for data analysis

We used separate dictionaries to identify which components of the MEF, taxonomic groups, tracking devices, software tools, or statistical methods were used in each paper. A dictionary is composed of concepts and associated words. For the MEF, the concepts of interest were the components of the framework (i.e. internal state, external factors, and motion and navigation capacities), and their associated words were terms potentially used in the abstracts to refer to the study of each component. For example, terms like ‘memory’, ‘sensory information’, ‘path integration’, or ‘orientation’ were used to identify the study of navigation. We used regular expressions to account for terms that could be prefixes or suffixes of larger words or, on the contrary, exact matches. Regular expressions were also used to account for conditions ‘and’, ‘or’, or ‘not’; e.g. for navigation, ‘sensory acquisition’ OR ‘sensory information’.

To assess how well each dictionary identified the concepts of interest, a quality control procedure was established. For each dictionary, a random sample of papers was selected, and a coauthor who did not lead the construction of that dictionary was randomly selected to check if those papers were correctly classified (i.e. accuracy). If the accuracy was less than 0.80, the dictionary would have to be modified and tested. The quality control process would be repeated until reaching an accuracy higher than 0.80. The size of the examined random sample was 100 papers for MEF, taxonomy, and devices dictionaries, and 50 papers for software and statistical methods. Devices, software, and methods dictionaries required attentive reading of title, abstracts, keywords, and M&M sections. While devices were considered relatively easier to identify, reading for software required correctly spotting mentions of ‘R’ or ‘Python’ as a software—to mention just a few examples. Statistical methods required more effort and domain expertise too, as there can be a diversity of methods mentioned in multiple parts of these sections. To avoid reviewer fatigue which would diminish the quality of our assessment, we reduced the sample of papers to review to 50 for software and statistical methods. The composition of the dictionaries, as well as their quality control results, are described in the following sections.

#### Taxonomic identification

To identify the taxonomy of the organisms studied in the papers, the Integrated Taxonomic Information System database (USGS Core Science Analytics and Synthesis) was used to detect names of any animal species (kingdom Animalia) that were mentioned in the abstracts, titles, and keywords. We screened these sections for latin and common (i.e. vernacular) names of species (both singular and plural), as well as common names of higher taxonomic levels such as orders and families. We excluded ambiguous terms that are part of latin or common names but also have a current language meaning; for example: ‘Here’, ‘Scales’, ‘Costa’, ‘Ray’, etc. Because we wanted to consider humans as a separate category, we used the following non-ambiguous terms to identify papers that focus on movement of humans (e.g. ‘player’, ‘patient’, ‘child’, ‘people’, ‘student’, ‘fishermen’, ‘person’, ‘hunter’, ‘runner’, ‘participant’, ‘athlete’). For the complete list of words, see Additional file 1: Sect. 3.2 at https://rociojoo.github.io/mov-eco-review/analyses.html#taxonomical-identification. In order to avoid cases where words may be suffixes of larger words, we used regular expression notation to match exact words, e.g ‘man’ must match only the word ‘man’ and not ‘manually’. We excluded words that could have an ambiguous meaning; e.g. ‘passenger’ may appear in papers that mention passenger pigeons, and ‘driver’ may be used to refer to a causing factor.

After having identified any taxon mentioned in a paper, we summarized taxa at the clade level, except for superclasses Osteichthyes and Chondrichthyes which we merged into a single group labeled fish, and for groups within the phylum Mollusca and the subphylum Crustacea which we considered collectively. Thus, each paper was classified as focusing on one or more taxonomic group, using the same nomenclature as [35]: amphibians, birds, crustaceans, fish, insects, mammals, mollusks, reptiles, and others (other invertebrates). For the purpose of our analysis, we kept humans as a separate category and did not count them within mammals. The code for taxonomic identification can be found in the Additional file 1 at https://rociojoo.github.io/mov-eco-review/R/taxonomy_analysis.R. The accuracy of the dictionary was 0.93.

#### Tracking devices for data collection

For this study, we considered any tool used for tracking animal movement to be a tracking device. These were grouped in 12 broad categories, that were meant to be as mutually exclusive as possible:

- *Accelerometer* Any technology that is placed on a subject and measures the acceleration of the tag, thus gathering information on the multidimensional movement of the individual which can be used to infer behavioral modes and energy expenditure.
- *Acoustic* Any technology that uses sound to infer location, either in a similar way to radio telemetry or in an acoustic array where animal vocalizations are recorded and the location of sensors in the array are used to obtain an animal’s location.
- *Body conditions* Any technology that uses body condition sensors to collect data on the subject that may be associated with a movement or lack thereof, such as temperature and heart rate.
- *Camera* Any device that records location/presence via camera; mainly camera traps with known locations where the capture of the individual implies the location.
- *Encounter* Any analog tracking method where the user must capture and place a marker on a subject (e.g. pit tags, bands). The recapturing/resighting of the subject infers the movement. This category was difficult to resolve in our study due to a lack of specificity in the phrases used in the literature.
- *GPS* Any technology that uses GPS satellites to calculate the location of an object, which can be handheld GPS devices or animal-borne tags.
- *Light loggers* Any technology that records light levels and derives locations based on the timing of twilight events.
- *Pressure* Any technology that records pressure readings to infer vertical movement, such as through a water column.
- *Radar* Any technology that uses ‘radio detection and ranging’ devices to track objects. It can be large weather arrays or tracking radars.
- *Radio telemetry* Any technology that infers location based on radio telemetry (VHF/UHF frequency).
- *Satellite* Any tag that collects location via, and sends data to, satellites (except Global Position System [GPS] satellites), so that data can be accessed remotely. The most frequently used system is ARGOS.
- *Video* Any device that records movement via video.

The use of one technology does not rule out the use of another technology; e.g. a combination of radio telemetry and GPS is commonly used in terrestrial movement studies. Thus, a paper can be counted in more than one of these categories. The dictionary of tracking devices is available in the Additional file 1 at https://rociojoo.github.io/mov-eco-review/Data/Dictionary/csv-updated-versions/Dictionaries-Data.csv and its accuracy was 0.84.

#### Software for data processing and analysis

Based on expert opinion, we compiled a list of 33 software packages, which use we evaluated: (1) Agent-Analyst, (2) BASTrak, (3) Biotas, (4) C (C code written by the researchers), (5) databases (any relational database, likely for data management and summarizing as needed for analytical use), (6) e-surge, (7) Fortran, (8) fragstats, (9) Genstat, (10) GME (Geospatial Modeling Environment), (11) GRASS, (12) Java, (13) m-surge, (14) MARK (program Mark and not the R packages RMark or unmarked), (15) Mathcad, (16) Matlab, (17) Microsoft Excel, (18) Noldus observer, (19) PAST, (20) PostGIS (we separated PostGIS from the database category because of its high spatial analytical capabilities), (21) Primer-e, (22) Python, (23) QGIS, (24) R, (25) SAS, (26) SPSS, (27) STATA, (28) Statistica, (29) Statview, (30) u-care, (31) UCINET, (32) Vicon, and (33) WinBUGS. The dictionary with the terms used is available in the Additional file 1 at https://rociojoo.github.io/mov-eco-review/Data/Dictionary/csv-updated-versions/Dictionaries-Software.csv. The accuracy of the dictionary was 0.88.

#### Statistical methods to analyze and model movement

We investigated the use of statistical methods in the movement literature with a similar dictionary approach. We first used expert opinion to compile all known statistical methods (and terms used for them) that could have been used to study movement, resulting in 188 terms; see the full list of terms in the Additional file 1 at https://rociojoo.github.io/mov-eco-review/Data/Dictionary/Methods-Classification.csv. Though we might have missed some terminology, we tried to be as exhaustive as possible. There is no unique way to classify statistical methods. For the purpose of this study, we classified them into the following 6 categories:

- *Generic* Generic statistical methods that could be used in any type of study, that are not inherently spatial, temporal, or social (e.g. a regression analysis)
- *Movement* Statistical methods used for the study of movement (e.g. behavioral change point analysis, [36])
- *Social* Statistical methods that are not exclusively for movement, but that characterize or model social processes (e.g. social networks)
- *Spatial* Spatial statistical methods (e.g. geostatistics)
- *Spatiotemporal* Spatiotemporal but not movement methods (e.g. spatiotemporal geostatistics)
- *Time-series* Time series methods (e.g. functional data analysis)

Terms related to hypothesis-testing (e.g., ‘t-test’) were considered in a preliminary list of terms but ultimately removed; we considered that the tendency of papers to present *p* values could be biasing researchers towards the use of hypothesis testing for publication acceptance, thus creating a bias towards general methods. The accuracy of the methods dictionary was 0.84.

To identify the most popular methods, a frequency analysis for sequences of three consecutive words, called trigrams, was also performed in the M&M sections of papers. Trigrams that did not correspond to statistical methods (e.g. ‘development core team’, ‘methods study site’) were manually discarded. Trigrams were chosen instead of bigrams because the latter often did not have enough words to provide information on a particular type of method. This was not an exhaustive analysis but provided us with a first approximation to method use and popularity.

#### The movement ecology framework

To assess the study of the different components of the MEF, we created a dictionary based on the descriptions of these components in [1]:

- *Internal state* The inner state affecting motivation and readiness to move. Terms used included regular expressions of: age class, body mass, breeding or reproductive stage, endogenous, energy intake, heart rate, hormonal, hunger, intrinsic factor, morphology, neurological, personality, physiological, psychological, and telomere, among others.
- *External factors* The set of biotic and abiotic environmental factors that affect movement. Terms used included regular expressions of: abiotic, biotic, conspecific, diel, environmental factor, extrinsic factor, group behavior, habitat, human disturbance, landmark, precipitation, oceanography, prey distribution or availability, sea surface temperature, social interaction, storm, temperature, topography, vegetation, and weather, among others.
- *Motion* The set of traits enabling the individual to execute movement. Terms used included regular expressions of: ballistic, biomechanic, locomotion, flapping, kinematics, random walk, running, soaring flight, swimming, tortuosity, and walking, among others.
- *Navigation* The set of traits enabling the individual to orient. Terms used included regular expressions of: chemoreception, cognition, compass, homing, path integration, magnetoreception, memory, olfaction, sensory information, and spatial, among others.

The dictionary of the MEF is available in the Additional file 1 at https://rociojoo.github.io/mov-eco-review/Data/Dictionary/csv-updated-versions/Dictionaries-Framework.csv. The accuracy of the dictionary was 0.91.

To look for changes in 2009–2018 with respect to the decade before, we identified movement papers from 1999 to 2008 using the algorithm described in the Data collection section and applied the same MEF dictionary to their title, abstract, and keywords.

### Topic analysis

The topics covered in the articles were treated as a latent variable, i.e. not directly observed, since articles do not always explicitly enumerate all the topics they cover. We assumed that the abstracts contained all the necessary information about the topics covered, and that an abstract could cover one or more topics. For that reason, we fitted a Latent Dirichlet Allocation (LDA) model to the abstracts to identify the hidden topics. LDAs are Bayesian mixture models that assume the existence of a fixed number *K* of topics behind a set of documents (i.e. the abstracts). Each topic can be characterized by a multinomial distribution of words with parameter $\beta$, drawn from a Dirichlet distribution with parameter $\delta$. Each document $d \in {1, ..., D}$ is composed by a mixture of topics, drawn from a multinomial distribution with parameter $\theta$, which is drawn from a Dirichlet distribution with parameter $\alpha$. For each word *w* in document *d*, first a hidden topic *z* is selected from the multinomial distribution with parameter $\theta$. From the selected topic *z*, a word is selected based on the multinomial distribution with parameter $\beta$. The log-likelihood of a document $d = \{w_1,...,w_N\}$ is $l(\alpha ,\beta ) = \log \int \sum _z\left[ \prod _{i=1}^{N} p(w_i|z_i,\beta )p(z_i|\theta )\right] p(\theta |\alpha )d\theta$

The model assumes exchangeability (i.e. the order of words is negligible), that topics are uncorrelated, and that the number of topics is known. Here we used the LDA model with variational EM estimation [37, 38] implemented in the topicmodels package. All the details of the model specification and estimation in general are in [39], while the description of data preprocessing and model fitting for this study are in Additional file 1: Sects. 3.1.2 and 3.1.3. The most commonly used criterion to choose a number of topics is the perplexity score or likelihood of a test dataset [40]. This quantity measures the degree of uncertainty a language model has when predicting some new text. However, the number of topics with the minimum perplexity score do not guarantee obtaining actual humanly-interpretable latent topics [41]. In fact, using this score could result in too many topics (e.g. in [42] abstracts were analyzed and 300 topics were obtained). For this study, we evaluated the perplexity score for 5, 10, 15, 20, 25, 30, 40, and 50 topics, and its value always decreased with more topics. However, 20 topics were already too many to interpret, label, and easily distinguish from the others, and 10 were too general, rendering them difficult to interpret as well. We thus fixed the number of topics at 15.

To interpret the topics, we used the posterior expected values of the word distribution per topic $E(\beta | z,w)$, denoted by ${\hat{\beta }}$. The words with the highest ${\hat{\beta }}$ values would be the most important ones in the topic. To facilitate the visualization of these ${\hat{\beta }}$ values, we created wordclouds for each topic, where the area occupied by each word was proportional to its ${\hat{\beta }}$ value and only words with ${\hat{\beta }} > 0.003$ were displayed. The posterior expectation of each topic per abstract $E(\theta _d | z)$, denoted by $\gamma$, indicated the degree of association between each abstract and each topic. We used $\gamma$ to identify the abstracts with the strongest association to each topic and used then as complementary information for the interpretation of the topics. With the aid of the wordclouds and highest-$\gamma$ abstracts, we labeled the topics. Topic analysis is analogous to k-means in the sense that the relationships between the latent groups and the observed variables are used by the researcher for interpretation and labeling, and that, while there can be some individuals (here abstracts) laying on the edges of a group, labeling is mostly based on the most representative cases. The sum of $\gamma$ values for each topic ($\sum _d E(\theta _d | z_k)$ for each topic *k*) served as proxies of the ‘prevalence’ of the topic relative to all other topics and were used to rank them.

We expected each abstract to be strongly associated to few topics. For a visual inspection of consistency with this expectation, we created a heatmap of the $\gamma$ values per abstract and topic (see Fig. 3.8 in Additional file 1: Sect. 3.1.4). To check for consistency within each topic, we compared its wordcloud with one obtained from abstracts that were highly associated with the topic ($\gamma > 0.75$). The two wordclouds should be telling a very similar story, thus visually resemble, with very small differences due to the abstracts being composed—in a small proportion—by other topics as well (see Fig. 3.9 in Additional file 1: Sect. 3.1.5.1 and more details in the same section). To assess the interpretability of the topics, we performed a word intrusion analysis: we asked 10 researchers in the field to identify a word injected into the top terms of each topic. We then computed the number of correct answers for each topic. A high score for a topic would indicate that the topic was easy to interpret using the four highest associated words to it. More details and results of the word intrusion analyses are in Additional file 1: Sect. 3.1.5.2.

## Results

### Data collection and processing

A total of 8007 papers were obtained for the period 2009–2018 (Fig. 1, Table 2). The proportion of animal and human movement papers over the total number of scientific papers extracted from WoS was higher in the last years (Table 2).

**Table 2:** Number of articles in movement ecology of animals and human mobility as identified by our algorithm, and articles in scientific literature in general, published from 2009 to 2018 according to the Web of Science

Year	Movement articles	All articles	Proportion movement/all ($10^{-4}$)
2009	485	1139611	4.25
2010	479	1186928	4.03
2011	564	1262956	4.47
2012	666	1323677	5.03
2013	791	1398009	5.66
2014	878	1438134	6.11
2015	978	1709898	5.72
2016	937	1775745	5.28
2017	1073	1838351	5.84
2018	1156	1928507	5.99

Proportion movement/all refers to the proportion of articles that studied movement

We downloaded the articles we had access to as xml or pdf documents, comprising a total of 4060 complete manuscripts, representing 51% of our list of movement papers. Then, we were able to extract 3674 Material and methods sections, which corresponded to 46% of all the papers and 90% of the fully downloaded papers; not all papers had an M&Ms section (e.g. reviews or perspective papers).

### Taxonomic identification

**Fig. 3:**
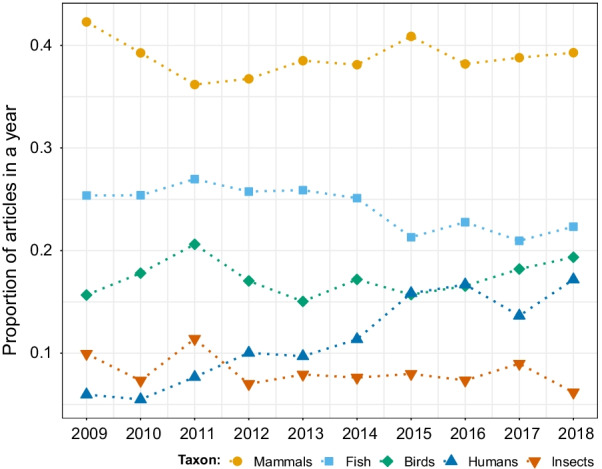
Proportion of articles in movement ecology of animals and human mobility studying each of the five most commonly studied taxonomic groups from 2009 to 2018. Mammals refer to other than humans. A study can investigate several taxonomic groups, hence the proportions for each year can sum up to more than one. See alt-text in the Alt-text section of the manuscript

**Fig. 4:**
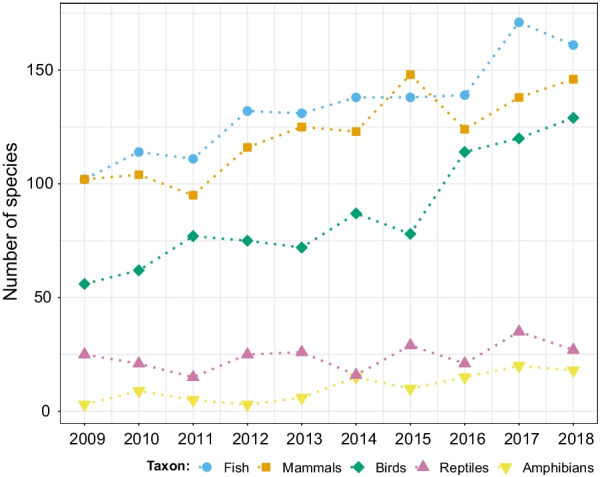
Number of different species studied in each year for the five taxonomic groups with most studied species in movement ecology of animals and human mobility from 2009 to 2018. See alt-text in the Alt-text section of the manuscript

Mammals were the most studied taxonomic group (33% of the papers where we found associations with any organism), followed by fish (20%), birds (15%), humans (11%), insects (7%), reptiles (6%), other invertebrates (3%), crustaceans (3%), mollusks (2%), and amphibians (2%). While mammals and fish were consistently the most studied groups in each year, the percentage of studies associated to humans showed an increasing trend, even matching the number of studies of birds in 2015 and 2016 (Fig. 3). Mammals, fish, and birds were also the three groups with the most studied species, and they showed an overall increasing trend in the number of species studied over the years (Fig. 4).

### Tracking devices for data collection

**Fig. 5:**
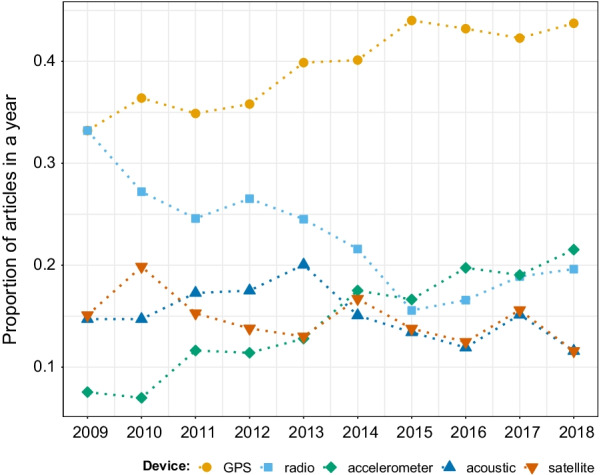
Proportion of articles in movement ecology of animals and human mobility using the five most commonly used tracking devices from 2009 to 2018. A study can use more than one device, hence the proportions for each year can sum up to more than one. See alt-text in the Alt-text section of the manuscript

The five most used tracking devices during 2009–2018 were GPS (28% of the papers), radiotelemetry devices (15%), accelerometers (11%), acoustic telemetry (10%), and satellite technology (10%). GPS did not only remain the most popular device in movement studies, but its popularity in relation to other methods increased throughout the years (Fig. 5). While in 2009 radio telemetry was as popular as GPS, GPS seems to have increasingly replaced radio telemetry [43].

On the other hand, there was an increase in the popularity of accelerometers (Fig. 5), which allow for research on movement in three-dimensional space, as well as the identification of fine-scale behaviors and the calculation of energy expenditure.

### Software for data processing and analysis

**Fig. 6:**
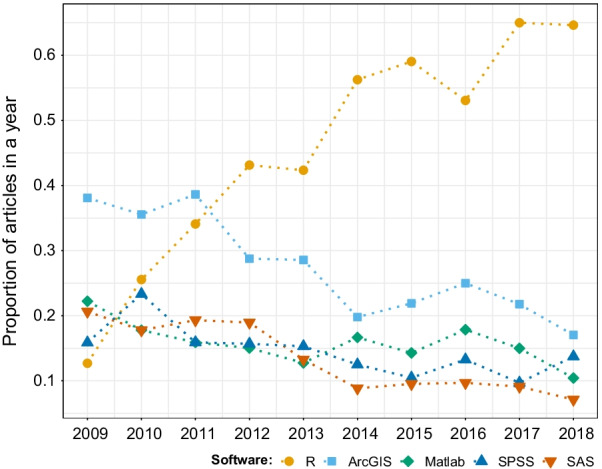
Proportion of articles in movement ecology of animals and human mobility using the five most commonly used software from 2009 to 2018. A study can use more than one software, hence the proportions for each year can sum up to more than one. See alt-text in the Alt-text section of the manuscript

The increasing volume and diversity of movement data obtained through tracking devices require appropriate software tools for data management, processing, and analysis [28, 44]. The most frequently mentioned software over the study period were R (38%), ArcGIS (18%), Matlab (11%), SPSS (10%), and SAS (8%). All the other software reached less than 3% of the papers with identified mentions of software. Among those, R experienced a constant and strong growth during the last 10 years while usage of all other software substantially decreased, making R an undisputed preference in the field (65% of prevalence in 2018, the last year of our study; Fig. 6).

### Statistical methods to analyze and model movement

**Fig. 7:**
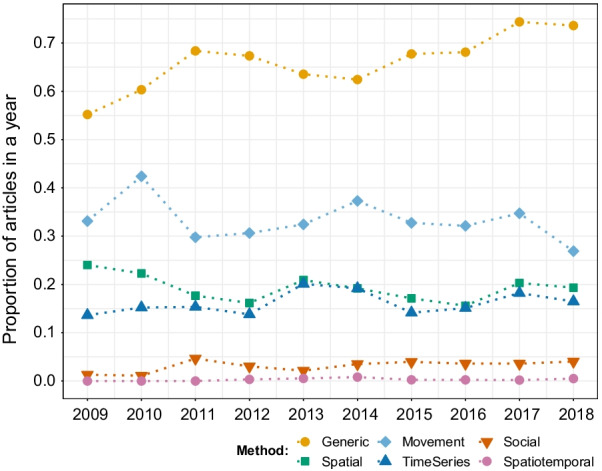
Proportion of articles in movement ecology of animals and human mobility mentioning each type of statistical method from 2009 to 2018. A study can use more than one type of method, hence the proportions for each year can sum up to more than one. See alt-text in the Alt-text section of the manuscript

Most studies (68%) used generic statistical methods (i.e. with no explicit spatial, temporal or social interaction component in its definition), showing an increasing trend in popularity (Fig. 7). Fewer studies used at least one or more specialized methods, i.e. movement (33%), spatial (19%), time series (17%), social analysis methods (3%), or non-movement spatiotemporal (0.3%). Our analysis reveals that researchers are not necessarily using movement-specific techniques to analyze movement, and, in some cases (42%), not using spatial, temporal, or social methods either.

In a trigram analysis, we assessed the frequency of all groups of three consecutive terms in the M&M sections of papers. The most popular statistical trigrams were related to mixed models (e.g. linear mixed models, linear mixed effects). Two movement trigrams, correlated random walks and hidden Markov models, were mentioned more than 100 times (Table 3).

**Table 3:** Most common statistical trigrams in M&M sections of articles in movement ecology of animals and human mobility from 2009 to 2018

Trigram	n
Linear mixed models	231
Linear mixed effects	229
Generalized linear mixed	202
Mixed effects models	202
Linear mixed model	188
Markov chain monte	180
Chain Monte Carlo	178
Akaike’s information criterion	174
Akaike information criterion	162
Minimum convex polygon	158
Information criterion aic	146
Monte Carlo mcmc	133
Correlated random walk	129
Mixed effects model	117
Hidden Markov model	116

Only trigrams with more than 100 mentions are listed

### The movement ecology framework

**Fig. 8:**
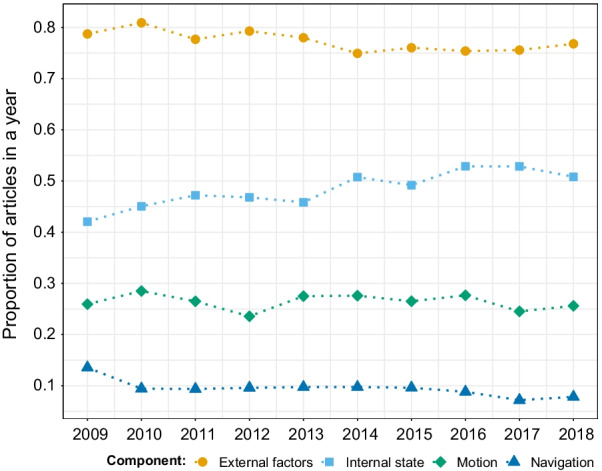
Proportion of articles in movement ecology of animals and human mobility focusing on each component of the Movement Ecology Framework from 2009 to 2018. A study can focus on more than one component, hence the proportions for each year can sum up to more than one. See alt-text in the Alt-text section of the manuscript

We found that, during 2009–2018, most studies tackled movement in relation to external factors (77%), while a minority of them studied the three other components (49%, 26%, and 9%, for internal factors, motion, and navigation capacity, respectively). In particular, there appeared to be a slight decrease in the number of studies focusing on navigation (Fig. 8). Of the 77% of papers investigating external factors, the majority studied the relationship between movement and the environment (80%), while movement associated with other animals, anthropogenic effects, and others were studied in 38%, 14%, and 3% of the papers, respectively (papers could have studied more than one factor so the percentages do not sum up to 100%).

For a more holistic understanding of movement, some studies attempted to incorporate multiple MEF components into their analysis. Half of the papers (51%) looked at more than one component; half of these (25%) tackled external factors and internal state together. For example, a study tracked Scopoli’s shearwaters (*Calonectris diomedea*) with GPS and accelerometers and fishing vessels with GPS to study the effect of the presence of the vessels on seabird behavior, particularly on their foraging effort and ultimately nutritional gain [45]. Each of the other combinations of components were studied by less than 10% of the papers (Table 4). Less than 1% of the papers looked at all four components of the MEF together. For example, a study quantified the effects of wind speed, wind direction, ambient temperature, and sun position on damselfly movement, orientation, and activities (including foraging and engaging in territorial interactions with competitors) [46]. The author found a relationship between the abiotic conditions and some activities, and evidence of rheotaxis presumably to minimize biomechanical costs when flying and foraging. As such, this work addressed the influence of internal state, external factors, motion capacity, and navigation capacity on damselfly movement. In a theoretical study that also jointly evaluated all four components of the MEF, the process by which individuals move between patches was explored by constructing a continuous-space population model with individuals carrying heritable trait values that affected the parametrization of their biased correlated random walks, resulting in individuals orienting towards suitable habitats [47]. This work presented a theoretical and simulation framework to test evolutionary hypotheses about the movement of organisms taking into account environmental factors, internal state, orientation, and dispersal patterns.

**Table 4:** Number and percentage of articles in movement ecology of animals and human mobility studying each combination of components of the Movement Ecology Framework for the decades 2009–2018 and 1999–2008

External	Internal	Motion	Navigation	2009–2018		1999–2008	
				Count	Percentage	Count	Percentage
X	–	–	–	2371	33.3	418	33.8
X	X	–	–	1768	24.8	287	23.2
–	X	–	–	663	9.3	96	7.8
X	–	X	–	485	6.8	104	8.4
X	X	X	–	424	6.0	69	5.6
–	–	X	–	383	5.4	56	4.5
–	X	X	–	373	5.2	63	5.1
X	–	–	X	176	2.5	44	3.6
X	X	–	X	136	1.9	15	1.2
–	–	–	X	83	1.2	23	1.9
X	–	X	X	73	1.0	12	1.0
–	X	–	X	50	0.7	16	1.3
–	–	X	X	49	0.7	15	1.2
X	X	X	X	45	0.6	11	0.9
–	X	X	X	41	0.6	8	0.6

In each row, an X in the column indicates the studied component

**Fig. 9:**
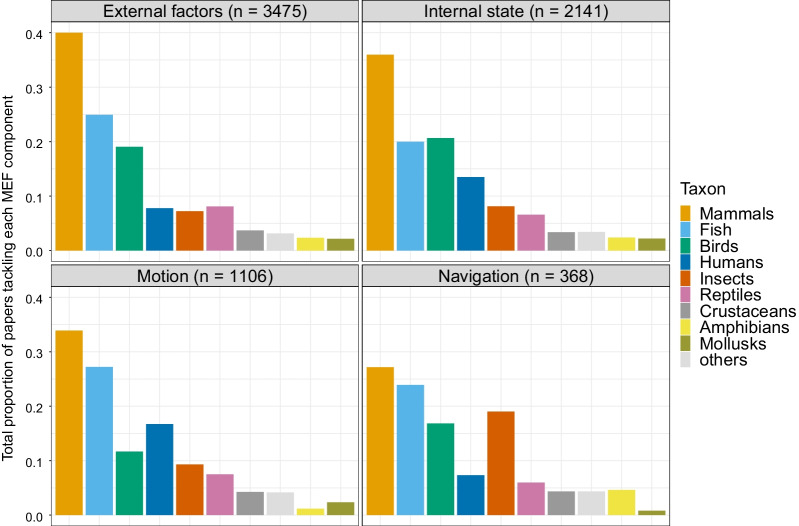
Proportion of articles in movement studying each taxonomic group for each component of the Movement Ecology Framework from 2009 to 2018. Humans were extracted from mammals. Articles associated to several components were accounted for in each relevant frame. See alt-text in the Alt-text section of the manuscript

Numerous studies on insects have focused on navigation (Fig. 9)—mostly in controlled environments—and their insights on cognitive mechanisms to navigate have inspired navigational models and studies in other taxa [48]. Motion has been an important focus of human mobility studies and could inspire animal motion research, particularly when accounting for interactions between individuals [49–51].

We found the same overall pattern in papers from 1999 to 2008, with no notable change: half of the papers (52%) looked at more than one component, a majority of studies investigated external factors (77%), and a minority of them studied the three other components (46%, 27%, and 12%, for internal factors, motion, and navigation capacity, respectively); research on internal state showed a small growth (from 46 to 49% in the most recent decade). The percentage of papers associated with each combination of components was also similar to the 2009–2018 decade (Table 4); only those involving external factors and the internal state slightly increased (Table 4). In retrospect, the terms “random walk” and “tortuosity” might pertain more to navigation than to motion, or else do not match any component as they are often used in the context of the movement path, an emergent property of the MEF. Removing these keywords from motion and adding them to navigation resulted in a lower percentage of papers addressing motion (from 26% to 24% in 2009–2018 and 28% to 25% in 1999–2008) and higher for navigation (from 9% to 12% in 2009–2018 and 11% to 14% in 1999–2008). Regardless, our overall conclusions are not sensitive to the classification of these particular terms.

### Topics covered

**Fig. 10:**
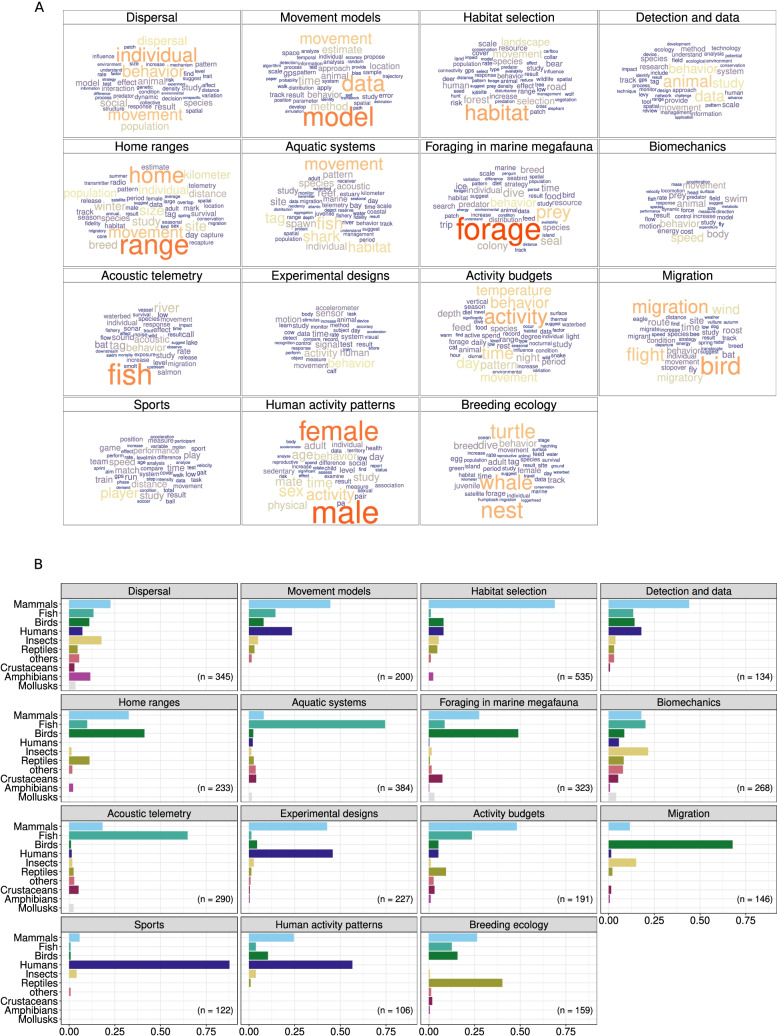
Topic analysis. **A** Worclouds of each topic based on ${\hat{\beta }}$ values. The area occupied by each word is proportional to its ${\hat{\beta }}$ value. Only words with ${\hat{\beta }} > 0.003$ are displayed. **B** For each topic, relative frequencies of papers studying each taxonomical group (humans were separated from mammals). Only papers with more than 50% of association to each topic (${\gamma }$) and with taxonomy information were used for this graph (n in the top right corner of each topic graph). While topics like ‘Sports’ and ‘Human activity patterns’ are mainly human-related, a few papers related to them took approaches from animal studies to study humans and a few others did the opposite; e.g. analyses inspired in sport journals to investigate animal performance in terms of speed or distance covered. See alt-text in the Alt-text section of the manuscript

Based on the LDA model outputs—mainly the terms that were highly associated with each topic (Fig. 10)—the topics covered were labeled as follows (in descending order of prevalence):

1. *Dispersal*, at individual, group, and population levels and spread across all taxa.
2. *Movement models*, encompassing any type of model (e.g. generalized linear model, random walks, agent-based models) that could be used to study dynamics, patterns, and populations, mostly in mammals including humans (68% of the total, from which 24% were humans).
3. *Habitat selection*, which encompasses choices in space use, influenced by resource availability or risks (e.g. natural predators or human disturbance), mostly in terrestrial mammals (69%; e.g. bears, wolves, deer, caribou, and elephants).
4. *Detection and data*, focused on the collection of movement information and the required technological devices. This topic is also mainly related to mammal studies (incuding humans), but also fish and birds.
5. *Home ranges*, mostly focused on the identification of areas where animals live and carry out their activities, and the geographical extent of this area, and mainly featuring mammals and birds, and to a lesser extent, reptiles.
6. *Aquatic systems*, involving the study of aquatic species (mostly fish; 74%), their migration, reproductive behavior and habitat use, often for management purposes.
7. *Foraging in marine megafauna*, consisting of foraging strategies and behavior of marine top predators, mostly seabirds (e.g. penguins) and marine mammals (e.g. seals).
8. *Biomechanics*, focused on body motion, swimming or flight power, and kinematics across most taxa.
9. *Acoustic telemetry*, used to monitor animal movement, or in some cases, effects of anthropogenic noise on animal behavior, mostly for fish (e.g. salmon) but also mammals (e.g. bats).
10. *Experimental designs*, which involve analyzing behavioral and movement responses based on multiple stimuli, mostly on humans and domestic animals (e.g. cattle).
11. *Activity budgets*, investigating—mostly using telemetry data—the effect of environmental conditions on the time allocated to different activities across temporal cycles (e.g. diel, seasonal), mostly in mammals (e.g. cats), fish and reptiles (e.g snakes).
12. *Migration*, encompassing migration routes, orientation and flight strategies, mostly in birds (e.g. eagles and vultures), but also some mammals (e.g. bats).
13. *Sports*, consisting of motion analysis of human sports players for better performance.
14. *Human activity patterns*, mostly related to health and physical activity in humans, including sex differences, and often sampled with accelerometers.
15. *Breeding ecology*, involving space use and movement corridors during breeding seasons; mostly, but not exclusively on sea turtles and whales.

## Discussion

### Reviewing literature with text mining and comparison with other approaches

With a rate of $\sim 3$ new papers published per day, animal and human movement literature is growing at a fast pace, which required quantitative approaches for an extensive review of the state of the art. The search algorithm performed well at identifying movement papers (0.90 precision). The set of papers analyzed in this study was not a complete list of animal and human movement papers; only 69% of the papers in Movement Ecology were part of our dataset (although this value could be partly explained by the fact that we did not include literature on plant movement ecology in our search). However, we have a very large sample of animal and human movement literature, with extensive keywords aiming to be as representative and precise as possible.

Aiming to gather and review papers about the movement of organisms or gametes, WoS was also used in [35] to build their literature dataset. They had a two-step criterion to select the papers. First, they screened Web of Science for papers that contained a list of 13 keywords (combined in a certain way; Table 1 and Additional file 1 Text A in [35]) to identify potential journals. Then, two of the coauthors narrowed down the selection to a list of 496 journals by excluding from their initial results those not ‘likely to contain articles relevant to ecology or evolution that addressed organismal movement’. Among the remaining journals, they selected a random sample of 1000 papers using a larger list of  70 words. Aiming for a precision of at least 0.60, these rules resulted in a precision of 0.77. The authors also referred to an estimate of 0.65 that would be analogous to the sensitivity estimation we did for this work. They also mentioned that their choice of keywords was ‘somewhat’ biased against microorganism movement. Though the scope of their work was different than ours (our search was focused on movement from Animalia only), we were inspired by their work, and used some of their terms as starting points in the algorithm. A more recent quantitative review of movement ecology literature and its use for conservation [52] also used WoS with a list of only 6 words in abstract, title, or keyword. The authors searched for ‘ecology’ and either ‘movement’, ‘migrat’, ‘home range’, ‘dispersal’ or ‘track’. They did not mention any performance statistic (e.g. precision, specificity, sensitivity).

This review, like [35, 52], only integrated articles in English, making the implicit assumption that these are representative of the research done in movement ecology of animals and human mobility. In our case, constraining the search to English facilitated the analysis since there are many more tools for Natural Language Processing in English than in any other language. The development of tools to process and analyze text in multiple languages would allow for future studies that would take into account a greater diversity in movement research.

Another bias common to all the reviews (ours, [35, 52]) came from the choice of search engine. We were not able to get papers that were not in WoS, which depends on WoS agreements with publishers and the institutions we accessed it from. In addition, the possibility to download articles was conditioned by openness of data from the publishers and data & text mining agreements with the institutions we accessed them from.

The dictionary analyses showed high precision at identifying the papers associated with each MEF component, taxonomic group, tracking device, software, and type of statistical method (0.91, 0.93, 0.84, 0.88, and 0.84 accuracy, respectively). Only [35] analyzed some of these dimensions (MEF components and taxonomic groups), and did so by manual examination of their sample of 1000 papers (the abstracts were read, and only when deemed necessary, the full documents were read too). To our knowledge, no other work in the literature of movement ecology or movement in general has attempted an automated approach to download scientific papers and extract the *Methods* section—or any other section except for tables.

The topic analysis showed general consistency (see Additional file 1: Sect. 3.1.5.1). The word intrusion approach is not an exhaustive assessment of topic interpretability, but it allows putting our results into perspective: some topics have a clear and easy interpretation and some others are really hard to interpret. In ecology, topic models have been recently used to identify themes in ecology [53] and assess the relationship between conservation biology and ecology literature [54], though interpretability tests like word intrusion were not mentioned. To our knowledge, this is the first movement review using a topic modeling approach and we hope that future reviews can adapt and improve this approach.

Each stage of the text mining approach (identifying movement papers, dictionary analyses, and topic modeling) assumed that there were no changes in the terms used in the literature to refer to the same concepts over the decade 2009–2018. While this assumption is likely to be false, it would be reasonable to expect that there have not been drastic changes in terminology within a single decade. We recommend that future studies also embrace text mining techniques, since the number of publications and the rate of publications are only expected to increase. For studies encompassing a longer time frame, it could be more useful to use the methods and criteria described here as a starting point—rather than the exact keywords or model parameters—and either train the algorithms over a decade and validate over others, or train a different algorithm for each time period. In any case, the validation process, which requires manual verification of a sample of papers, is key to support the findings; here, they returned high precision for identifying movement papers and high accuracy for each dictionary. ﻿In retrospect, we also encourage using version control when calibrating algorithms and keywords to keep track of the development process and get better performance in a more efficient way.

### Technology as the main driver of movement research

In the article introducing the MEF [1], technology and methods to quantify the movement of individuals were mentioned as main limitations to apply the proposed framework. Here, we showed that devices such as GPS and accelerometers, that can provide movement data at a high resolution, have become more commonly used. This is likely due to the development of cheaper, smaller, and more efficient devices, which are now feasible options for small and medium-sized animals [18]. These technological improvements are likely responsible for the increase in the number of species tracked (Fig. 4). Along with accelerometers, other loggers like magnetometers and gyroscopes—that are newer to movement ecology [55] and are becoming widespread [29]—are opening avenues to exploring physiological aspects of movement like energy expenditure [56]. These devices are also useful for uncovering behaviors at fine spatiotemporal resolutions, further contributing to the understanding of movement [29, 57, 58].

Our results showed a steady growth in the use of R. The same pattern in reported R usage was observed in the field of ecology globally [59]. According to both [59] and this study, the popularity of R in the late 2000s was low (used by $\sim 10\%$ of the papers), while the majority of articles published in the most recent years have reported its use, indicating a homogenization of movement and ecology towards R.

In parallel, there has been substantial progress in the number and sophistication of quantitative methods for the study of movement [27, 60] and the development of R packages to make these more accessible [28]. While the number of movement methods in the literature is increasing [28, 29], the proportion of papers using movement-specific analytical methods did not show the same pattern (Fig. 7). On the one hand, not all studies require movement-specific methods; the choice of methods should depend solely on the research question, assumptions, and data. On the other hand, movement is a complex process, and—in most cases—statistically noisy, nonlinear, and spatially and temporally correlated [61], calling for dedicated methods. Ideally, experts in methodological tools (e.g. statisticians) should work closely with data or field experts (e.g. ecologists) to choose, develop, and apply adequate methods to the research questions and the objects of study.

The results obtained here were consistent with the perspectives of movement ecologists who answered a limited survey in the winter and spring of 2019 (see Additional file 1: Sect. 4 at https://rociojoo.github.io/mov-eco-review/survey-about-movement-ecology.html). Respondents (32 out of 33) indicated that technology and methods to collect and analyze data were the features that revolutionized the field in the last decade—this has been coined the ‘biologging revolution’ [29] and the ‘data-driven revolution’ [26]. In most participants’ opinions (31 out of 32), current and future technological advances (e.g. ICARUS technology www.IcarusInitiative.org, accelerometers, multisensor loggers) will also be driving the field in the following decade.

### Opportunities for an improved understanding of movement

In the past decade, technology and methods have substantially reduced some major limitations to our understanding of movement. However, quantitatively, not much has changed in the study of the MEF in 2009–2018 with respect to the decade before (Table 4). More than three quarters of the studies still investigate the set of biotic and abiotic environmental factors that affected the movement of individuals (alone or combined with one or two other components of the MEF), while less than 1% of studies examined all four components together (Table 3).

While it is logical that researchers explicitly study or control for the effects of the environment (including other individuals), we would have expected to find an increase in the study of the internal state, motion, and navigation, as well as in the percentage of studies addressing other components of the MEF in combination with external factors. Research on internal state showed a small growth over the years, which could be due to an increase in the number of studies investigating the links between energetic conditions and behavior within tracks [21], or the increase in the studies examining the links between individual behavioral type (i.e personality) and movement [62]. Thus, the technological improvements have not been sufficiently exploited.

Addressing all of the components of the MEF requires interdisciplinary efforts involving researchers from ecology, biology, neuroscience, physics, statistics, and geographic information science, among others [29, 35]. These efforts would benefit from bridging the divide between human mobility research and animal movement ecology, and between aquatic, terrestrial, and aerial realms. There are already virtuous examples of good interdisciplinary research in movement ecology; for instance, studies bringing together concepts and theory from animal and human movement [27], or exploring the use of geophysical phenomena as auditory cues for animal navigation [63].

Overall, the topic analysis revealed both the fragmentation of the movement ecology community based on taxonomic groups, and the potential for synergies across taxonomic groups. In particular, we were able to quantitatively categorize topics clearly associated to specific taxa, notably in the aquatic realm (one topic about aquatic systems in general, and three about foraging, acoustic telemetry and breeding ecology of marine species). Humans (two topics about sports and activity patterns) and birds (one topic about migration) were also strongly associated to given topics, further reinforcing a pattern of study of large, easy to tag fauna that work as model species. On the other hand, the majority of topics did not show fidelity to any taxon, which suggests their potential for generalization—and integration—across taxa, for instance using shared methods or devices. Among these, two groups of topics can be identified, dealing with subject areas (dispersal, habitat selection, home ranges, biomechanics, and activity budget) and technological and methodological concerns (movement models, detection and data, experimental designs), both of which lay the foundations for further interdisciplinary research and knowledge transfer.

Integrated research also requires overcoming communication difficulties [64, 65], developing structures that encourage interdisciplinarity in our institutions [66], and engaging in movement-research networks (e.g. the European COST Action ‘MOVE—Knowledge Discovery from Moving Objects’ [67], ENRAM—The European Network for the Radar surveillance of Animal Movement [68], or the International Biologging Society [69]). Intra-institutional support and inter-institutional networks could be important pillars to overcome the communication challenges and the difficulties of obtaining funding for interdisciplinary research [70].

The progress made in terms of tools for data collection, processing, and analysis needs to be shared with the community to foster a better and more integral understanding of movement in all of its aspects. In this respect, the FAIR principles (Findable, Accessible, Interoperable, and Reusable) [71, 72] offer useful guidelines to make all data, methods, and software publicly available and shared.

By congregating the community around the R environment, most movement researchers can communicate over the same programming language, share codes, and move towards transparency, collaboration, and reproducibility. In an era with large volumes of data and much dependency on software for the analyses, reproducibility in science also requires open code [73]. On﻿ the other hand, the dominance of R in the field should not prevent researchers from using tools to process or analyze movement in other software (e.g. movingpandas [74] and scikit-mobility [75] in Python). Conveniently, integrated development environments like RStudio or Jupyter allow for running codes in several programming languages (e.g. R and Python).

## Conclusions

Improvements in technological devices to track animals and humans have generated high volumes of movement data from a range of species, providing greater information on their movement paths, physiology, and the environment they experience. However, there has been little change in the degree to which studies address different components of the MEF, while there are also distinct groups of research topics that are predominantly linked to the species studied (e.g. aquatic versus terrestrial), the methods used and their application. Developments in statistical methods and software tools have facilitated data processing and analysis. These aspects have been clear drivers of movement research in the 2009–2018 decade and will likely continue to drive the field, allowing to explore new research questions and improve our understanding of evolutionary, physiological, and life-history causes and consequences of movement. To make that possible, there should be strong commitments towards transparency, reproducibility, and interdisciplinary collaboration practices in the community.

## Additional Files

Additional file 1—Companion website: This is the companion website for the manuscript, https://rociojoo.github.io/mov-eco-review/, serving as the manuscript’s Supplementary Information page. It contains an Introduction (to the website) and the abstract (section 1), a description of Data collection and processing (section 2), Data analysis with a description of methods and layout of results (section 3), the description and results of the Survey about movement ecology (section 4), and details on the R session used for these analyses. The R codes are available in a GitHub repository https://github.com/rociojoo/mov-eco-review, and links to specific R codes are provided in the text of the website.

## Alt-text for Figures

**Fig. 1:** Time line of movement articles, with the number of articles published in the y-axis, and the years in the x-axis. There is an increasing trend in the number of articles going to almost 1200 papers in 2018. In this time line, we also highlighted events in the history of movement studies: The philosophical study of movement (Aristotle's De Motu Animalium; ~325 BCE), the mechanistic study of movement (Galen's De Motu Musculorum; ~170 BCE), Quantitative analysis of movement (Turchin; 1998), the introduction of movement as a framework (Nathan et al. 2008), a Biotelemetry and biologging issue in JAE, and a movement ecology special feature in PNAS (both in 2008), the GPS in animal ecology theme issue in PTRSB (2010), the start of the Movement Ecology journal (2013), the movement ecology virtual issue in JAE (2015), and the collective movement ecology issue in PTRSB (2018).

**Fig. 2:** Graphical representation of the algorithm to identify movement papers. First, define KEYWORDS for Web of Science and search. Get results on N papers. Then do quality control, consisting of selecting a random sample n (n = 100) from N, reading the abstracts, determining if they are from movement studies and computing a precision metric. If precision is greater than 0.8 and N is greater than 5000, we stop. Else, we edit keywords and do it all over again.

**Fig. 3:** Proportion of papers in each year studying each of the five most commonly studied taxonomic groups. The years are in the x-axis, the proportion of papers in each year in the y-axis, and dots of different colors (one per class) correspond to the value of proportion for each year. In all years from 2009 to 2018, mammals (orange) were the most studied group, followed by fish (sky blue) , birds (green), humans (blue), and insects (dark orange). Only from 2012 there were more studies concerning humans than those of insects.

**Fig. 4:** Number of species studied in each year for the five classes with most studied species (fish, mammals, birds, reptiles, and amphibians, in that order). More species have been studied in the last years in general.

**Fig. 5:** Proportion of papers in each year using the five most commonly used tracking devices. The years are in the x-axis, the proportion of papers in each year in the y-axis, and dots of different colors (one per device) correspond to the value of proportion for each year. GPS (orange) showed an increasing trend and was the most used device in movement papers in all years. Radio telemetry (sky blue), showed a decreasing trend, and started in 2009 with the same proportion of studies as GPS, and ended in 2018 in third place, below accelerometer (green). In contrast, accelerometer was in fifth position in 2009 and increased its popularity over the years. Acoustic telemetry (yellow) and satellite technology (blue) were tied in fourth place in 2018.

**Fig. 6:** Proportion of papers in each year using the five most commonly used software. The years are in the x-axis, the proportion of papers in each year in the y-axis, and dots of different colors (one per software) correspond to the value of proportion for each year. R (orange) showed an increasing trend, starting at the last position of the five in 2009, and ending in first place in 2018 (with 0.7 of studies using it). The other four software showed decreasing trends over the years. ArcGIS (sky blue), SPSS (yellow), Matlab (green), and SAS (blue), ended up in second, third, fourth, and fifth positions, respectively, in 2018.

**Fig. 7:** Proportion of papers in each year mentioning each type of statistical method. The years are in the x-axis, the proportion of papers in each year in the y-axis, and dots of different colors (one per type of method) correspond to the value of proportion for each year. Generic methods (orange) were the most popular throughout 2009-2018, while movement (sky blue), spatial (green), time series (yellow), social (blue), and spatiotemporal (dark orange), remained in second, third, fourth, fifth, and sixth position, respectively, throughout the decade.

**Fig. 8:** Proportion of papers in each year focusing on each component of the MEF. The years are in the x-axis, the proportion of papers in each year in the y-axis, and dots of different colors (one per component) correspond to the value of proportion for each year. In all years from 2009 to 2018, external factors (orange) were the most studied component, followed by internal state (sky blue), motion (green), and navigation (blue).

**Fig. 9:** Bar plots of the proportion of papers studying each taxonomical group for each component of the MEF. The number of papers where taxonomical groups were identified by our algorithm for each component are indicated in the figure: 3475 for external factors, 2141 for internal state, 1106 for motion, and 368 for navigation. In all components, mammals were the most studied. For external factors, fish and birds were the second and third most studied groups, respectively. For internal state, they were birds and fish, in that order. For motion, fish and humans. And for navigation, fish and insects, followed closely by birds.

**Fig. 10:** Topic analysis. A: Word clouds of each topic based on beta values. The area occupied by each word in each wordcloud is proportional to beta. In topic 1, the largest words are individual, behavior, movement, dispersal, population. In topic 2, model, data, movement, time, estimate, method. In topic 3, habitat, movement, landscape, selection. In topic 4, animal, data, behavior, study. In topic 5, range, home, movement, site, size, population, individual, kilometer. In topic 6, movement, fish, habitat, tag, shark. In topic 7, forage, prey, behavior, dive, seal. In topic 8, speed, behavior, animal, movement, swim, body. In topic 9, fish, behavior, tag, river, acoustic. In topic 10, behavior, human, signal, sensor, activity, motion. In topic 11, activity, behavior, time, temperature, day. In topic 12, bird, migration, flight, migratory, wind. In topic 13, player, distance, play, speed, train. In topic 14, female, male, sex, activity, behavior, sex, age. In topic 15, turtle, whale, nest, behavior, breed, adult. B: Bar plots of proportion of papers studying each taxonomical group for each topic. Regarding dispersal (n = 345 papers), the most studied groups were mammals, human and amphibians. For movement models (n = 200), they were mainly mammals and humans. In habitat selection (n = 535), most studies pertained mammals. In detection and data (n = 134), also a majority of mammals. In home ranges (n = 233), it was mostly birds, followed by mammals. In aquatic systems (n = 384), an overwhelming majority of fish. In foraging in marine megafauna (n = 323), it was mostly birds, followed by mammals. In biomechanics (n = 268), insects, fish and mammals were the most studied groups. In acoustic telemetry (n = 290), fish were the most studied, followed by mammals. In experimental designs (n = 227), humans and mammals were the most studied groups. In activity budgets (n = 191), mammals were the most studied, followed by fish. In migration (n = 146), birds were the most studied group. In sports (n = 122), almost all studied involved humans. In human activity patterns (n = 106), it was humans, followed by mammals. In breeding ecology (n = 159), it was mostly reptiles, followed by mammals, birds, and fish.

## Data Availability

We provide complete R code and details, from descriptions of word search on WoS and scripts to download the papers, up to the codes to reproduce the plots in this manuscript, in a companion website, publicly hosted at https://rociojoo.github.io/mov-eco-review. The website works as the online Supplementary Information to this manuscript. The repository also contains a dataset of movement papers obtained after pre-processing the query results from WoS and applying the cleaning procedure. The entire digital content is archived in a public Zenodo repository: https://doi.org/10.5281/zenodo.4024706. The authors can be directly contacted for further development and questions about the full datasets that include the published articles, which have not been released to respect text and data mining rights of the publishers.

## Abbreviations

DOI: Digital object identifier
FAIR: Findable, accessible, interoperable, and reusable principles
LDA: Latent Dirichlet allocation
M&M: ﻿Materials and methods
MEF: Movement ecology framework
SI: Supplementary information
WoS: Web of Science
